# Preliminary Assays towards Melanoma Cells Using Phototherapy with Gold-Based Nanomaterials

**DOI:** 10.3390/nano10081536

**Published:** 2020-08-05

**Authors:** Joana Lopes, João Miguel Pinto Coelho, Pedro Manuel Cardoso Vieira, Ana Silveira Viana, Maria Manuela Gaspar, Catarina Reis

**Affiliations:** 1 Research Institute for Medicines (iMed.ULisboa), Faculty of Pharmacy, Universidade de Lisboa, Av. Professor Gama Pinto, 1649-003 Lisboa, Portugal; joanamargaridalopes@campus.ul.pt; 2 Instituto de Biofísica e Engenharia Biomédica (IBEB), Faculdade de Ciências, Universidade de Lisboa, Campo Grande, 1749-016 Lisboa, Portugal; jmcoelho@fc.ul.pt; 3 Departamento de Física, Faculdade de Ciências e Tecnologia, Nova University of Lisbon, 2825-149 Caparica, Portugal; pmv@fct.unl.pt; 4 Centro de Química Estrutural, Faculdade de Ciências, Universidade de Lisboa, Campo Grande, 1749-016 Lisboa, Portugal; apsemedo@fc.ul.pt

**Keywords:** melanoma, gold nanoparticles, bioproduction, laser photothermal therapy, in vitro models, experimental results

## Abstract

Cancer like melanoma is a complex disease, for which standard therapies have significant adverse side effects that in most cases are ineffective and highly unspecific. Thus, a new paradigm has come with the need of achieving alternative (less invasive) and effective therapies. In this work, biocompatible gold nanoparticles (GNPs) coated with hyaluronic acid and oleic acid were prepared and characterized in terms of size, morphology and cytotoxicity in the presence of *Saccharomyces cerevisiae*, and two cell lines, the keratinocytes (healthy skin cells, HaCat) and the melanoma cells (B16F10). Results showed that these GNPs absorb within the near-infrared region (750–1400 nm), in the optical therapeutic window (from 650 to 1300 nm), in contrast to other commercial gold nanoparticles, which enables light to penetrate into deep skin layers. A laser emitting in this region was applied and its effect also analyzed. The coated GNPs showed a spherical morphology with a mean size of 297 nm without cytotoxic effects towards yeast and tested cell lines. Nevertheless, after laser irradiation, a reduction of 20% in B16F10 cell line viability was observed. In summary, this work appears to be a promising strategy for the treatment of non-metastatic melanoma or other superficial tumors.

## 1. Introduction

Cancer is now one of the largest, if not the largest, public health problem, accounting for nearly 13% of worldwide deaths [1]. To highlight the magnitude of this problem, it is estimated that until 2035 the global annual incidence of cancer may double compared to the current one, which could reach about 29.4 million new cases [2]. The incidence of melanoma is rising, faster than almost any other type of cancer [3]. This year, 2020, only in the United States, 1,806,590 new cancer cases is estimated to occur and 100,350 will be due to melanoma resulting in a high number of deaths [4].

Melanoma can be described as a malignant tumor originated from melanocytes, present in the lower part of the epidermis (basal cell layer), usually having a black or brownish color because melanocytes do not stop producing melanin [5,6]. Ninety five percent of melanomas are located at the skin level, cutaneous melanoma, and there are still a small percentage (5%) that develop in the mucosa, retina and meninges [5,7].

The early detection of melanoma is one of the key points of this disease prognosis, once in these cases the treatment is usually curative being that the 5-year relative survival rate is more than 90%. Moreover, eighty-four percent of cases are diagnosed at a localized stage [8]. It requires special attention for new or changing skin growths, new lesions and changes in the existent lesion’s appearance, based on the “ABCDE rule”, asymmetry, border irregularity, color variation, diameter < 5 mm and evolution of the lesion, or a sore that does not heal. The development of metastasis to other organs and body sites makes the prognosis severely worst. At advanced phases of the disease, the efficiency of the therapies is already compromised [9]. Nevertheless, more than half of the patients diagnosed with the distant-stage disease now survive for at least one year [8].

The causes of melanoma are not yet fully understood, but it is known that there are different internal and external risk factors. Internal factors include, namely, ethnicity, personal or family history, skin and light eyes, red or blonde hair, freckles and genetic conditions such as xeroderma pigmentosum. Other internal risk factors are the development of dysplastic nevi, immunosuppression and age. The incidence rate increases with an age up to about 60 years; however, it is also one of the most common cancers at younger ages [9,10,11]. External factors include, for example, exposure to ultraviolet radiation from sunlight, to occupational chemicals, or by the use of indoor tanning, being the last one classified as carcinogenic by the International Agency for Research on Cancer [8,9,10,11].

The number of therapies available for non-metastatic melanoma is increasing. Whenever possible, removal by surgery is the first-line option. However, adjuvant therapies are most often followed, such as chemotherapy, radiotherapy, immunotherapy and more recently targeted therapy [5]. Nonetheless, the efficiency of these available therapies is far from being satisfactory and your use is generally limited by their toxicity and serious side effects that reduce the patient’s quality of life [12]. However, coupled with the advancement of science, boosts the development of novel specific therapies, with increased efficacy and less side effects [13].

Over the last 30 years, many studies have demonstrated the efficacy of nanosized materials for tumor targeting, diagnosis and therapy [14,15]. Nanosized materials allow one to surpass the disadvantages of some conventional therapies. Nanoparticles (NPs) allow one to increase the circulation time of a drug into the bloodstream [16] and promote its accumulation at the tumor site due to their structure (actively using ligands onto the surface as an example) and small size due to their intrinsic capability to permeate through the blood vessels and accumulate in the tumor site. This last phenomenon is associated to the enhanced permeability and retention effect (EPR), which is due to the fact that tumor blood vessels have fenestrae and endothelial gaps with increased vascular permeability and in combination with a defect of lymphatic drainage, allows the accumulation of NPs and other nanosized material (20–100 nm) at the tumor environment [13].

In the other hand, the photothermal therapy (PTT) also has aroused the interest of many researchers, since it is a minimally invasive therapy rarely associated to complications, and enabling the patient to recover fairly quickly compared to existing methods [17,18]. After intratumoral administration, gold nanoparticles (GNPs) can be activated by increasing the local temperature. The GNPs activation occurs using a laser source where NPs are able to convert optical energy in thermal energy. NIR-lasers show an efficient response in killing cancer cells with less power than the visible-lasers operate [14]. This revolutionary therapy becomes advantageous mainly taking advantage of the near-infrared (NIR) region (from 750 to 1400 nm) of the optical therapeutic window (from 650 to 1300 nm), where the light can penetrate further in the tissue (lower absorption and scattering), and on the surface plasmon resonance (SPR) effect of the NPs, which highly increases the absorption of the light when compared with the surrounding tissues, and thus reducing any collateral damage or inflammation of nearby healthy tissues [17,18]. The latter effect depends on the size and shape of the nanoparticle material and surrounding medium. However, this therapy is limited to the depth capability of the laser irradiation and, therefore, it is mainly limited to skin cancers such as non-metastatic melanoma [14,17,19]. For this reason, it is so important to have a formulation of NPs that has a high absorption capacity at a specific desired wavelength, as is the case of the GNPs formulation developed in this work.

There has been much research around the GNPs; there are already several well-known and studied methods to produce GNPs, namely Turkevich’s method [20], Brust-Schiffrin’s method [21] and the Seed-mediated Growth Medium method [22,23]. However, most of all the supra-referenced methods involve the use of toxic chemicals, such as the quaternary ammonium salt, cetyl trimethylammonium bromide (CTAB), which is not at all compatible with the *in vivo* use [14].

Therefore, it is urgent to replace these highly toxic compounds by others with biocompatible properties.

**Figure 1:**
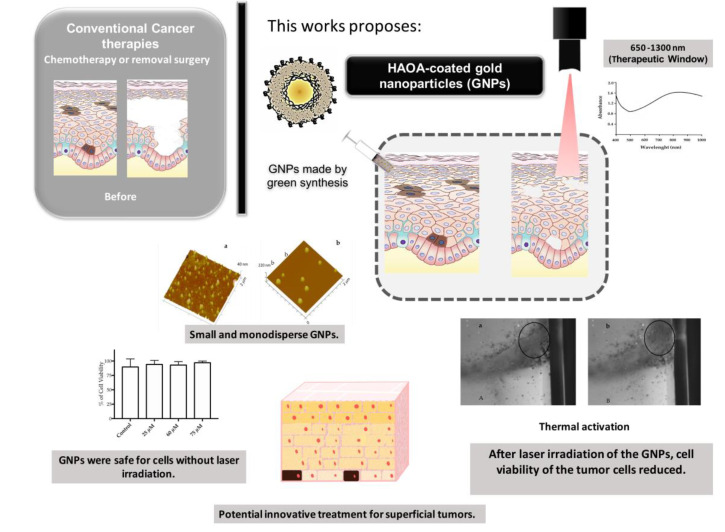
Schematic representation of the laser activation of HAOA-coated gold nanoparticles (GNPs). HAOA means hyaluronic acid and oleic acid.

Considering all background previously described, this work aims to characterize GNPs produced with the Modified Seed-mediated Growth method (without toxic compounds like CTAB) in terms of mean size, morphology and cytotoxicity, in the presence of *Saccharomyces cerevisiae*, and two cell lines, the human keratinocytes (HaCat) and the murine melanoma (B16F10). It was also important to know what wavelengths GNPs absorb and to access their behavior before and after laser activation. The aim is to have an absorption behavior of GNPs in the NIR zone, i.e., less energy and high deepness. As Supplementary Materials, this work also accessed the macroscopic behavior of GNPs using the phantom model that mimics the skin’s structure and laser as the light source. Figure 1 demonstrates the main objectives and results of the present work.

## 2. Materials and Methods

### 2.1. Materials

Gold (III) chloride trihydrate (HAuCl_4_), silver nitrate (AgNO_3_), L-ascorbic acid (L-AA), aqueous solution containing 5.9% of rosmarinic acid, hyaluronic acid (HA) from *Streptococcus Equi* (MW ~1.5–1.8 × 10E6 Da) and oleic acid (OA; MW: 282.46 gmol^−1^) were all supplied by Sigma-Aldrich (Steinheim, Germany). Methylene blue, Sudan III and the yeast *Saccharomyces Cerevisiae* were donated by Universidade Lusófona de Humanidades e Tecnologias (ULHT). The human keratinocyte cell line, HaCat, and the murine melanoma cell line, B16F10, were provided by Cell Line Service GmbH (Eppelheim, Germany). Culture media and antibiotics were obtained from Invitrogen (Life Technologies Corporation, Grand Island, NY, USA). Reagents for cell proliferation assays were purchased from Promega, (Madison, WI, USA). All the remaining chemicals and substrates used were of analytical grade. The water used was purified (18.2 MW × cm at 25 °C) through a Millipore system (Millipore, Burlington, MA, USA).

### 2.2. Methods

#### 2.2.1. Preparation of Gold Nanoparticles (GNPs)

Briefly, fresh solutions of gold salt, silver nitrate, L-ascorbic acid and an aqueous solution containing 5.9% of rosmarinic acid were prepared and concentrations were based on a previous work [14]. The coating was previously prepared by mixing hyaluronic acid (HA) and oleic acid (OA), and the so formed suspension was stirred overnight at 400 rpm and 60 °C.

The reaction for preparing uncoated GNPs was carried out for 15 min at 800 rpm in a stirring plate (Fisherbrand ARE Hotplate Stirrer, Bradford, UK). After 15 min, the coating suspension (HAOA) was added to the GNPs core (proportion 1:1, *v*/*v*) and the stirring continued overnight at 800 rpm at room temperature.

On the following day, the GNPs were stored at 4 °C, and protected from the light.

#### 2.2.2. Size, Polydispersity Index and Zeta Potential Measurements

Uncoated GNPs and HAOA-coated GNPs (diluted samples with water, 1:10) were characterized in terms of mean particle size and polydispersity index (PdI), using dynamic light scattering in a Coulter Nano-sizer Delsa Nano CTM (Fullerton, CA, USA) in 3 series of 11 measurements each analyzed sample. In addition, its zeta potential was also assessed by laser Doppler spectroscopy (ZetaSizer Malvern Instruments, Malvern, UK) in 3 series of 11 measurements each analyzed sample (diluted in NaCl at 0.1 M, 1:10).

#### 2.2.3. Atomic Force Microscopy (AFM)

Morphology of uncoated GNPs and HAOA-coated GNPs was measured. The samples were prepared by placing a drop (40 µL) of GNPs suspension on a freshly cleaved mica surface and left to dry before being analyzed. The images were acquired by atomic force microscopy (AFM) (Multimode 8 coupled to Nanoscope V Controller from Bruker, UK), using peak force tapping and ScanAssist mode. Tip model used was scanasyst-air 0.4 N/m, Bruker.

#### 2.2.4. Absorbance Spectrum

Maximum absorbance peak of uncoated GNPs (dilution 1:1) and HAOA-coated GNPs (without dilution) was determined conducting an absorbance spectrum, from 400 to 1000 nm, in a spectrophotometer (Shimadzu UV-160A, Shimadzu Europa GmbH, Duisburg, Germany).

#### 2.2.5. Cytotoxicity on the *Saccharomyces cerevisiae* Model

This model was chosen due to the similarity with mammalian cells, the yeast fast growth, the inexpensiveness and easy cultivation [24]. To carry out this model, *Saccharomyces cerevisiae* (ATCC^®^ 9763TM) was cultivated in Petri dishes containing 1.5% agar and a complete medium YPD (Yeast extract 1%, Peptone 0.5% and Dextrose 2%). After, *Saccharomyces cerevisiae* suspension was inoculated in an Erlenmeyer, containing 20 mL of YPD medium, and incubated at 30 °C without agitation for 24 h to obtain approximately 1 × 10^7^ cells/mL. The tested concentrations of HAOA-coated GNPs were 25, 60 and 75 μM suspended in YPD medium to achieve a final volume of 2 mL and placed in plastic cuvettes. A control assay without the GNPs with the same volume was also conducted. The cuvettes were incubated for 4 h, ensuring a minimum of 2 h of log growth phase (as it will be explained in the next Section 3 and Section 4), at 30 °C under stirring at 230 rpm (*n* = 4). Then, the number of cells per mL in each cuvette was calculated according to the following Equation (1) [24]:

(1) $$Y=6.8219\times 10^{-8}\;X+0.0327$$

where X is the number of cells per milliliter and Y is the absorbance of the samples.

Absorbance of samples was performed at 525 nm in a spectrophotometer (Thermo Scientific, model Evolution 300 BB, Loughborough, UK) at the predetermined times: 0, 1, 2 and 4 h after incubation.

#### 2.2.6. In Vitro Assays on HaCat and B16F10 Cell Lines

The cytotoxicity of HAOA-coated GNPs was evaluated in two cell lines, namely human keratinocytes (healthy cells, HaCat) and murine melanoma (B16F10) cells.

The objective of the first set was to select the GNPs concentration that do not affect the cell viability to further be used in laser irradiation tests. For this, both cell lines were incubated with different GNPs concentrations (5, 30 and 60 μM) for a period of 24 h. Additionally, it was also tested, separately, the effect of laser irradiation on the viability of the cell lines under study using GNPs at a concentration of 5 μM. In the laser assays, the loss of cell viability was accessed, combining the two factors, GNPs followed by laser activation. The GNPs were incubated with cells during 4 h before laser activation. The time of 4 h was based on a previous work done by our research group that even demonstrated that 1.5 h were sufficient for the internalization of this type of GNPs by cancer cells [25]. All the laser irradiation tests were performed for 3 min with an irradiance of 0.04 W/cm^2^.

For all assays, cells were grown in Dulbecco’s modified Eagle’s medium (DMEM) with glucose (4500 mg/L), supplemented with 10% inactivated fetal bovine serum and 100 IU/mL penicillin and 100 μg/mL streptomycin (Invitrogen; complete medium) at 37 °C under a 5% CO_2_ atmosphere. Maintenance of culture cells was performed every 2–3 days until cells reached a confluence of around 80%. For all cell lines, the medium was first removed from culture flasks and TrypLE Express was added for detaching adherent cells. After an incubation of 3 min, detached cells suspension was transferred to a Falcon tube and the complete medium was added. Finally, and after centrifugation for 10 min at 1000× *g* (Beckman Instruments, Inc., Brea, CA, USA), the medium was discarded and cells suspended in complete medium. Cells at a concentration of 5 × 10^4^ cells/mL (200 µL) were seeded in 96 well plates and allowed to adhere overnight. Cells were then incubated with the GNPs for 24 h or 4 h at 37 °C under a 5% CO_2_ atmosphere for the cytotoxicity and laser assays, respectively.

The cell viability was assessed using the 3-(4,5-dimethylthiazol-2-yl)-2,5-diphenyltetrazolium bromide (MTT) assay [26]. At the end of each in vitro assay, the cells’ medium was removed and cells washed with PBS. Then, MTT solution (50 µL) at a concentration of 0.5 mg/mL in the incomplete medium was added to all wells and plates were incubated for a 4 h period at 37 °C under a 5% CO_2_ atmosphere. After the incubation time, to solubilize the formazan crystals, DMSO (100 µL) was added to each well.

Then, plates were shacked for 10–15 min and absorbance was measured in a microplate reader at 570 nm (Model 680, Bio-Rad, Hercules, CA, USA).

The cell viability was evaluated by determining the percentage of viable cells (tested samples) related to control cells.

#### 2.2.7. In Vitro Thermal Activation Using the Phantom Model

In order to analyze the GNPs’ disruption, two types of colored dyes, the methylene blue (a hydrophilic dye) and the Sudan III (a hydrophobic dye) were exceptionally used. These two types of dyes were only used in order to macroscopically monitor the study of thermal action of GNPs presented in Section 3.3. of the results. They have different physicochemical properties in terms of water solubility. In both cases, the dye was at a concentration of 20 μL/mL and it was associated with GNPs during 24 h under stirring at 60 rpm. After staining, the HAOA-coated GNPs were lyophilized during 24 h at −50 °C (FreeZone 2.5 L Benchtop Freeze Dry System, Labconco, MO, USA). Besides this assay, these dyes will be not applied for further studies and in any clinical circumstances.

**Figure 2:**
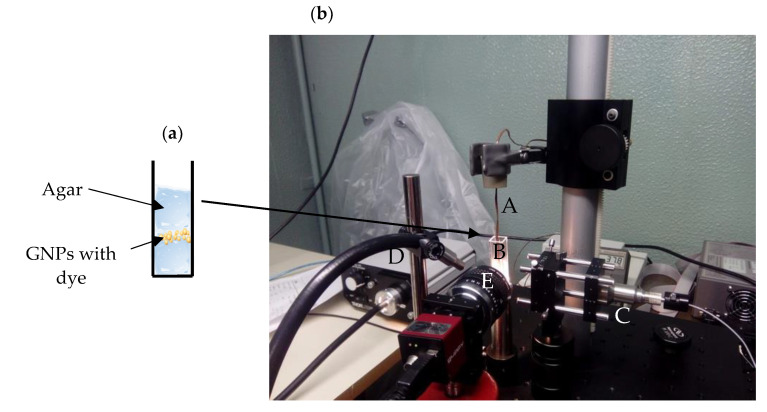
(**a**) Schematic representation of HAOA-coated GNPs included into the phantoms in cuvette and (**b**) irradiation apparatus. A—thermocouple, B—cuvette, C—laser delivery system, D—light source and E—digital camera.

The next step was the preparation of phantoms with agar 1% (*w*/*v*). Then, HAOA-coated GNPs were included into the phantoms, according to the scheme in Figure 2a. The release of the dye from the GNPs was observed by using a JDSU L4-2495-003 laser, emitting at a wavelength of 811 nm. The beam dimension at the target was optically controlled to a diameter of 6.7 mm. The effects of the irradiation were observed on a computer screen with the use of a digital camera (Pentax K50 18–55 WR) with a resolution of 640 × 480 at 64 fps. To improve the image contrast between some pre-existing coloration in the phantom (intrinsic color of GNPs with the dye) and the color due to dye release into the medium, black and white imaging was used.

The temperature was measured by placing a tip of a thermocouple in the cuvette as shown in Figure 2b. The optical power used for irradiation was between 1.75 and 2.87 W and the GNPs were heated until the measured temperature reached 39 °C.

#### 2.2.8. Statistical Analysis

All results are expressed as means ± standard deviation (S.D.). A one-way ANOVA analysis was applied to demonstrate statistical differences in all tested parameters.

## 3. Results

### 3.1. Physical and Optical Properties of GNPs

#### 3.1.1. Size, Polydispersity Index and Zeta Potential Measurements

The mean size and zeta potential of uncoated and coated GNPs are presented in the Table 1. As expected, an increase on the mean size for HAOA-coated GNPs was observed (297 nm versus 159 nm) when compared to uncoated nanoparticles. Regarding the zeta potential, it was observed a higher negative zeta potential of GNPs after HAOA coating (−19 mV versus −2 mV).

NPs size is a key parameter and it has a strong effect on their interactions with living cells, influencing uptake efficiency, internalization pathway selection, intracellular localization and cytotoxicity as when NPs enter in a biological environment, they find a wide variety of biomolecules [27]. Size is also important to take advantage of EPR effect since tumor tissues have an abnormal vascular nature. In addition, NPs mean size affect the SPR band, which is essential for the application of PTT. Although, the mean size is a critical factor, shape, surface charge and surface biochemistry are also important properties [27,28,29].

It is also desirable that NPs exhibit uniformity with respect to their size, thus presenting low PdI values [27,28,29]. It is noticed that uncoated GNPs exhibit a lower PdI (0.275) comparatively to HAOA-coated GNPs (0.438). After coating, the population of NPs formulation was less homogenous, probably because the volume of coating varies for each nanoparticle and chemical species involved. But, herein, larger GNPs (>100 nm) might be an additional advantage to maintain those GNPs in the target area. This last issue will be accessed in future assays. Concerning PdI values, it is generally assumed that values greater than 0.7 mostly indicate that the sample has a very heterogeneous size distribution. The various size distribution algorithms work with data that falls between these extremes. The calculations for these parameters are defined in the ISO 22412:2017 [30,31]. 

Finally, the uniformity of each produced batch is also a crucial factor for reproducibility and scale-up of this process. As complementary information, the DLS analysis performed on GNPs stored at 4 °C for three or six months after synthesis showed no change in the size of these nanoparticles, attesting to their reliable shelf life.

#### 3.1.2. Atomic Force Microscopy (AFM) 

**Figure 3:**
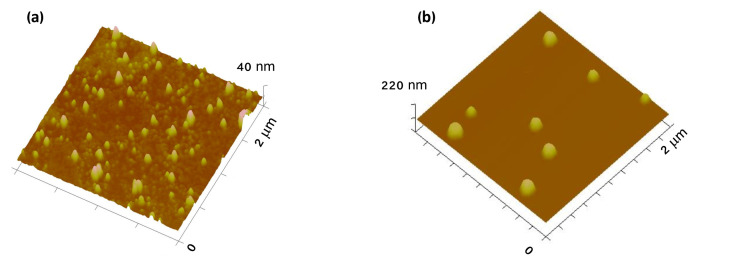
(**a**) 3D atomic force microscopy (AFM) images of uncoated GNPs and (**b**) HAOA-coated GNPs.

AFM images (Figure 3) show spherical and small GNPs, confirming the previous results obtained by the DLS technique. This is important as the SPR band is altered according to the shape of the particle [28]. As observed by DLS, an increase on HAOA-coated GNPs was achieved (Figure 3b).

#### 3.1.3. Absorbance Spectrum

**Figure 4:**
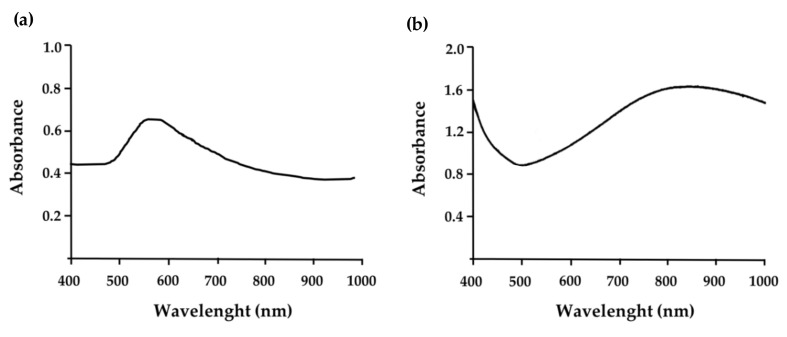
(**a**) Optical absorption spectrum of uncoated GNPs and (**b**) HAOA-coated GNPs, with a maximum absorption wavelength in the therapeutic optical window of biotissues (>750 nm), where absorption is lower.

The absorbance spectrum of uncoated GNPs (Figure 4a) revealed that the maximum absorbance peak was located around 560 nm. On the other hand, in HAOA-coated GNPs (Figure 4b), a shift for higher values of wavelength was observed with a maximum absorbance peak at approximately 850 nm (with low variation between 800 and 900 nm), confirming the previous expectations. This is a crucial parameter for the application of these HAOA-coated GNPs in PTT.

### 3.2. Cytotoxicity Studies of GNPs

#### 3.2.1. Evaluation on a *Saccharomyces cerevisiae* Model

**Figure 5:**
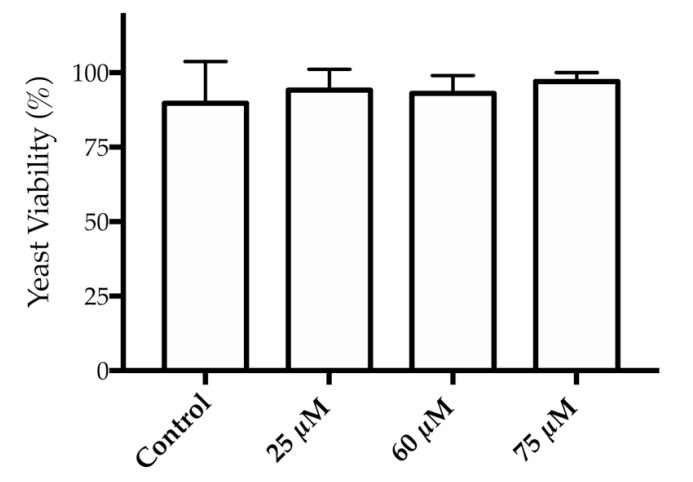
Yeast viability (%) after 4 h of incubation of HAOA-coated GNPs at 25, 60 and 75 µM (mean value ± S.D., *n* = 4).

The potential toxicity of the HAOA-coated GNPs was tested in a *Saccharomyces cerevisiae* model. According to Figure 5, it is observed that the incubation of HAOA-coated GNPs at concentrations ranging from 25 to 75 µM during 4 h was able to preserve the yeast viability.

#### 3.2.2. Evaluation on HaCat and B16F10 Cell Lines

The potential toxicity of the HAOA-coated GNPs was tested, by an MTT assay, on healthy skin HaCat cells and melanoma B16F10 cell lines. During the MTT assay, the 3-(4,5-dimethylthiazol-2-yl)-2,5-diphenyltetrazolium bromide compound (a yellow aqueous solution) is converted by mitochondrial succinate dehydrogenase to an insoluble colored (dark purple), formazan product being this product is directly proportional to the number of viable cells [32].

Regarding the results presented in Table 2, a decrease on cell viability of the murine melanoma cell line (B16F10) ranging from 84 to 67% was observed as GNPs concentrations increased from 5 to 60 µM. On the other hand, for HaCat cells, the panorama is quite different; as cell viability was always > 95% for all GNPs concentrations tested. Taking into account these results, the laser tests were performed using GNPs at concentration of 5 μM.

##### Evaluation on HaCat and B16F10 Cell Lines after GNPs’ Activation with Laser

The toxicity induced by the laser was also assessed; a slight reduction on the cell viability was observed as depicted in Table 3. The cellular viability in the presence of HAOA-coated GNPs at 5 µM was repeated in the same conditions.

Table 3 presents the percentage of cell viability combining GNPs incubation followed by laser activation. In immortalized human keratinocytes (HaCat), it was possible to observe that cell viability was maintained in comparison to cells submitted to laser irradiation. For B16F10, the murine melanoma cells, the viability of these cells was reduced by 20% (74% versus 94%) in comparison to B16F10 cells irradiated only with laser.

In this preliminary assay, it is observed that GNPs, when associated with a laser, had an additional effect on the loss of cell viability on melanoma cells’ viability, in contrast to healthy human keratinocytes. Moreover, this effect seems to be selective to melanoma cells, characterized by an overexpression of cell receptors to HA that is present in GNPs coating [33].

All these studies were performed after an incubation period of 4 h mimicking *in vivo* testing; results correspond to cell viability mean value ± S.D., *n* = 3.

### 3.3. Thermal Activation

**Figure 6:**
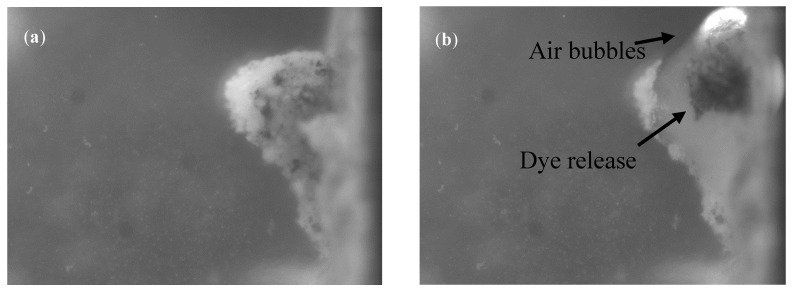
Results of thermal activation of HAOA-coated GNPs conjugated with methylene blue. (**a**) Before activation and (**b**) after activation where air bubbles and dye release are present (see more details in the video).

As referred previously, the HAOA-coated GNPs were conjugated with two colored dyes (Table 4). Methylene blue and Sudan III were tested relatively to their release after activation of GNPs with a laser as heat source (in all following figures, both dyes appeared as dark areas). It was possible to observe the GNPs’ disruption with consequent dye release (Figure 6) when the temperature of activation reached 39 °C, with the formation of air bubbles and movements of different intensity, including circular movements in all samples, with the exception of the blank that only contains agar. For higher detail, a video showing the thermal activation of HAOA-coated GNPs was included in the Supplementary Materials.

As previously referred, these GNPs are distinguished by their unique characteristics, such as SPR that is very suitable for therapeutic applications. When these GNPs are exposed to light, the oscillating electromagnetic field of the light induces a coherent oscillation of the free electrons (conduction band electrons) of the metal. This oscillation around the surface of the particle causes charge separation with respect to the ionic lattice, forming a dipole oscillation along the direction of the electric field of the light. The amplitude of this oscillation reaches a maximum at a specific frequency, called SPR. As mentioned earlier, this characteristic can be altered by size, shape, composition, among other factors characteristic of the NPs [28]. Our GNPs have a very suitable SPR for therapeutic applications as demonstrated by the maximum absorbance peak at approximately 850 nm (Figure 4b) in contrast to other commercial gold nanoparticles, which absorb at lower wavelengths, not allowing light to penetrate into deep skin layers.

## 4. Discussion

Several efforts are being made in research to obtain more targeted, effective and safe formulations for the treatment of melanoma, increasing the patient quality of life. Nanotechnology has demonstrated a high potential to treat cancers like melanoma. NPs can have a very important role, since they have the ability to reach cancer cells and penetrate into tumor vasculature, exerting the effect locally [34]. In this regard, PTT has been shown a promising strategy for treating superficial tumors. In this therapy, after injection at tumor sites, NPs are irradiated with a NIR laser converting the absorbed light into heat, leading to a highly localized hyperthermia that kill tumor cells [35,36]. In this area, some authors are using GNPs, which have unique physicochemical and optical characteristics, allowing the laser activation, as well as easy functionalization, good biocompatibility, large surface-to-volume ratio and facile synthesis [37].

In this study, it was described the production and characterization of HAOA-coated GNPs, using an alternative reducing agent, an aqueous solution containing 5.9% of rosmarinic acid. These GNPs showed a maximum absorbance wavelength between 800 and 900 nm, inside the therapeutic optical window of biotissues where absorption is lower and deeper tissues could be reached. The application of HAOA-coated GNPs in cancer therapy is possible because the high absorption peak means lower activation energy. For clinical use, it is now necessary to optimize the relationship between low activation energy and greater depth [14].

In this work, we also accessed the mean size and morphology of the produced GNPs because adequate mean sizes have to be fulfilled, as this will influence their in vivo fate [38]. According to the results obtained by DLS, after HAOA coating, GNPs showed an adequate particle diameter (297 nm) and PdI (below to 0.45). Curiously, the AFM results detected smaller particles that the ones detected by DLS. Through the AFM, it was also possible to observe that GNPs presented a spherical morphology. Herein, the spherical shape means high contact area versus volume, meaning high cellular interactions [39,40]. The size differences observed between the two techniques performed are even expected, since each methodoloy evaluates different properties of the sample. For example, in DLS, the hydrodynamic radius of the particles, including the ionic and solvent layers associated to the sample in suspension are measured, which does not happen in the AFM technique [41].

As already described, there are many methods to produce GNPs. Most of them result in GNPs smaller (below 100 nm) than those obtained in the present work, even when compared to uncoated ones [42]. The full optimization process was already described by our group in terms of the gold core but also in terms of the HAOA coating and the role of HA and OA [14,25]. However, we want to take advantage of the NPs mean size through the EPR effect. It is known that tumoral tissues have capillaries and blood vessels with larger pores, ranging from 100 to several hundred nanometers, compared with healthy tissues. So, NPs with sizes ranging from 100 to 500 nm, as the GNPs developed in the present work, can easily penetrate the tumor mass, in contrast with healthy tissues, characterized by an intact vasculature [43].

Regarding the activation tests of HAOA-coated GNPs with a laser, it was possible to observe the activation of NPs in all samples. This activation was translated on some movements inside the sample, including circular movements, comparable to convections movements and by the formation of air bubbles. The activation temperature was about 39 °C. Temperatures above 42 °C should be avoided because it might cause degradation of the muscle cells [44,45]. Moreover, due to their poor blood supply, tumors are more sensitive to heat compared to normal tissue, being selectively destroyed [46]. While the tests were conducted, one difficulty was related to the fact that it is necessary a perfect alignment between the laser and the NPs to be activated, which due to the dimensions involved and the invisible nature of the radiation, was a challenging task.

Regarding the mechanism that leads to the death of cancer cells, after selectively infiltrating malignant cells and being activated by the laser, it is believed that GNPs induce cell damage, such as protein denaturation and disruption of cell structure due to vaporization of the water inside the cell, which leads to the formation of small bubbles within the cell [44,45]. Then, the bubbles quickly expand and burst lysing the cancer cell.

Last but not least, the toxicological test in *Saccharomyces cerevisiae* and on HaCat and B16F10 cell lines was another key point in this study. In the *Saccharomyces cerevisiae* model, the GNPs did not demonstrate toxicity. At the higher concentration, they even promoted the growth of yeast as previously observed in our group with a different type of NPs [47]. It seems somehow that the polymer, HA, present in GNPs may be responsible for the yeast proliferation effect observed [48].

The selectivity of the GNPs throughout this work has not been assured by active targeting yet. However, in B16F10 cells, a higher cytotoxicity was observed compared to HaCat cells, which leads us to believe that GNPs developed in the present work are selective to tumor cells. It is probably due to the fact that melanoma cells express a greater number of CD44 receptors, which are natural ligands of HA that form part of the GNPs coating [33,49]. Thus, the two cell models used to assess toxicity are concordant and confirmed the non-toxicity of GNPs for healthy cells, before and after laser activation. However, further studies will have to be done to confirm these results including the validation in tumor murine models.

## 5. Conclusions

The HAOA-coated GNPs produced by the method described in the present work, displayed spherical morphology, small sizes and unique optical properties for thermal activation. GNPs absorb in the NIR range, enabling their activation with a laser as a heat source.

In the absence of laser, GNPs did not interfere with *Saccharomyces cerevisiae* and HaCat cell viability. In contrast, for B16F10 cells, an increase of GNPs concentration was translated to a decrease on cellular viability. Following GNPs activation by the laser, a reduction of 20% in B16F10 cell viability was observed, while in the human keratinocyte cell line, HaCat, a preservation on cellular viability was obtained.

Overall, these GNPs are a promising strategy to be applied for the treatment of superficial tumors. Further studies will include the cellular targeting studies, intracellular trafficking of GNPs and administration of the GNPs in a syngeneic murine melanoma model to validate their potential antitumor effect.

## Figures and Tables

**Table 1:** Mean particle size, polydispersity index (PdI) and mean zeta potential of uncoated GNPs and HAOA-coated GNPs. Data are present as mean ± S.D.

GNPs Formulation	Mean Size ± SD (nm)	Polydispersity Index (PdI)	Mean Zeta Potential± SD (mV)
Uncoated GNPs	159 ± 28	0.275	−2 ± 1
HAOA-coated GNPs	297 ± 3	0.438	−19 ± 3

**Table 2:** *In vitro* viability of B16F10 and HaCat cells 24 h after incubation with HAOA-coated GNPs (mean value ± S.D., *n* = 3).

HAOA-Coated GNPs Concentration	B16F10 Cells Viability (%)	HaCat Cells Viability (%)
5 μM	84 ± 5	95 ± 10
30 μM	77 ± 9	100 ± 5
60 μM	67 ± 5	109 ± 7

**Table 3:** Preliminary assays towards melanoma cells using phototherapy where the laser irradiation tests were carried out for 3 min.

Samples/Treatment	HaCat Cells	B16F10 Cells
	Cell Viability (%)	Cell Viability (%)
Only HAOA-coated GNPs at 5 μM	93 ± 10	88 ± 5
Only laser	88 ± 6	94 ± 4
HAOA-coated GNPs at 5 μM plus laser	86 ± 4	74 ± 6

**Table 4:** Phantom macroscopic aspect before and after laser activation using two experimental dyes (only used in this assay).

Dye	Phantom Macroscopic Aspect	Before Activation	After Laser Activation
Hydrophilic(Blue methylene)	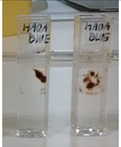	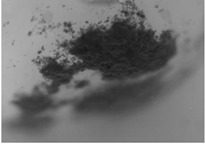	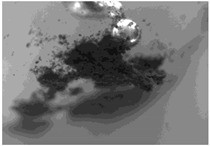
Lipophilic(Sudan III)	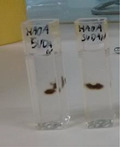	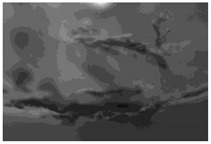	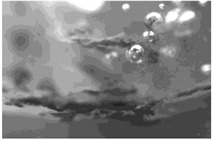

## Supplementary Materials

The following are available online at https://www.mdpi.com/2079-4991/10/8/1536/s1, Video S1: Thermal activation of HAOA-coated GNPs.
